# Osteoporosis in Rheumatoid Arthritis: Dangerous Liaisons

**DOI:** 10.3389/fmed.2020.601618

**Published:** 2020-11-23

**Authors:** Irene Llorente, Noelia García-Castañeda, Cristina Valero, Isidoro González-Álvaro, Santos Castañeda

**Affiliations:** ^1^Rheumatology Division, Hospital Universitario de La Princesa, IIS-IP, Madrid, Spain; ^2^Cátedra UAM-Roche, EPID-Future, Department of Medicine, Universidad Autónoma de Madrid (UAM), Madrid, Spain

**Keywords:** rheumatoid arthritis, bone erosions, inflammation, osteoimmunology, osteoporosis, RANKL/RANK/OPG, Wnt signaling

## Abstract

Osteoporosis has been classically considered a comorbidity of rheumatoid arthritis (RA). However, recent advances in the pathogenesis of osteoporosis in RA have shown a close interplay between cells of the immune system and those involved in bone remodeling, introducing new actors into the classic route in which osteoclast activation is related to the RANK/RANKL/OPG pathway. In fact, the inflammatory state in early stages of RA, mediated by interleukin (IL)-1, IL-6, IL-8 and tumor necrosis factor (TNF)-α has the ability to activate and differentiate osteoclasts not only through their relationship with RANKL, but also through the Wnt/DKK1/sclerostin pathway, leading to bone loss. The role of synovial fibroblasts and activated T lymphocytes in the expression of the RANKL system and its connection to bone destruction is also depicted. In addition, autoantibodies such as rheumatoid factor and anti-citrullinated protein antibodies are other pathogenic mechanisms for the development of bone erosions and systemic osteoporosis in RA, even before the onset of arthritis. The aim of this review is to unravel the relationship between different factors involved in the development of osteoporosis in RA patients, both the classic factors and the most novel, based on the relationship of autoantibodies with bone remodeling. Furthermore, we propose that bone mineral density measured by different techniques may be helpful as a biomarker of severity in early arthritis patients.

## Introduction

Rheumatoid arthritis (RA) is a systemic chronic inflammatory disease that primarily affects diarthrodial joints and is associated with disability, the presence of multiple comorbidities and decreased life expectancy (1). A recent cross-sectional epidemiological study estimates that the prevalence of RA in Spain is 1.07% [95% confidence interval (CI): 0.70–1.44] (2), similar to that described in western countries (1).

Osteoporosis (OP) is a frequent systemic skeletal disorder characterized by low bone mass and microarchitectural deterioration of bone tissue, resulting in bone fragility and susceptibility to fracture. Fragility fracture is defined as a spontaneous fracture that results from minimal or no identifiable trauma and represents a sign of OP (3).

The prevalence of OP in the general population ranges from 9 to 38% for women and 1 to 8% for men depending on the countries (3). In the European Union, it was estimated that 22 million women and 5.5 million men had osteoporosis in 2010 (4). A study calculated that the prevalence of global OP at the lumbar spine or femoral neck in Spanish female population was 12.7% according to densitometric criteria (5). More specifically, in women older than 50 years, prevalence was 22.8% at lumbar spine and 9.1% at femoral neck (5).

**Figure 1:**
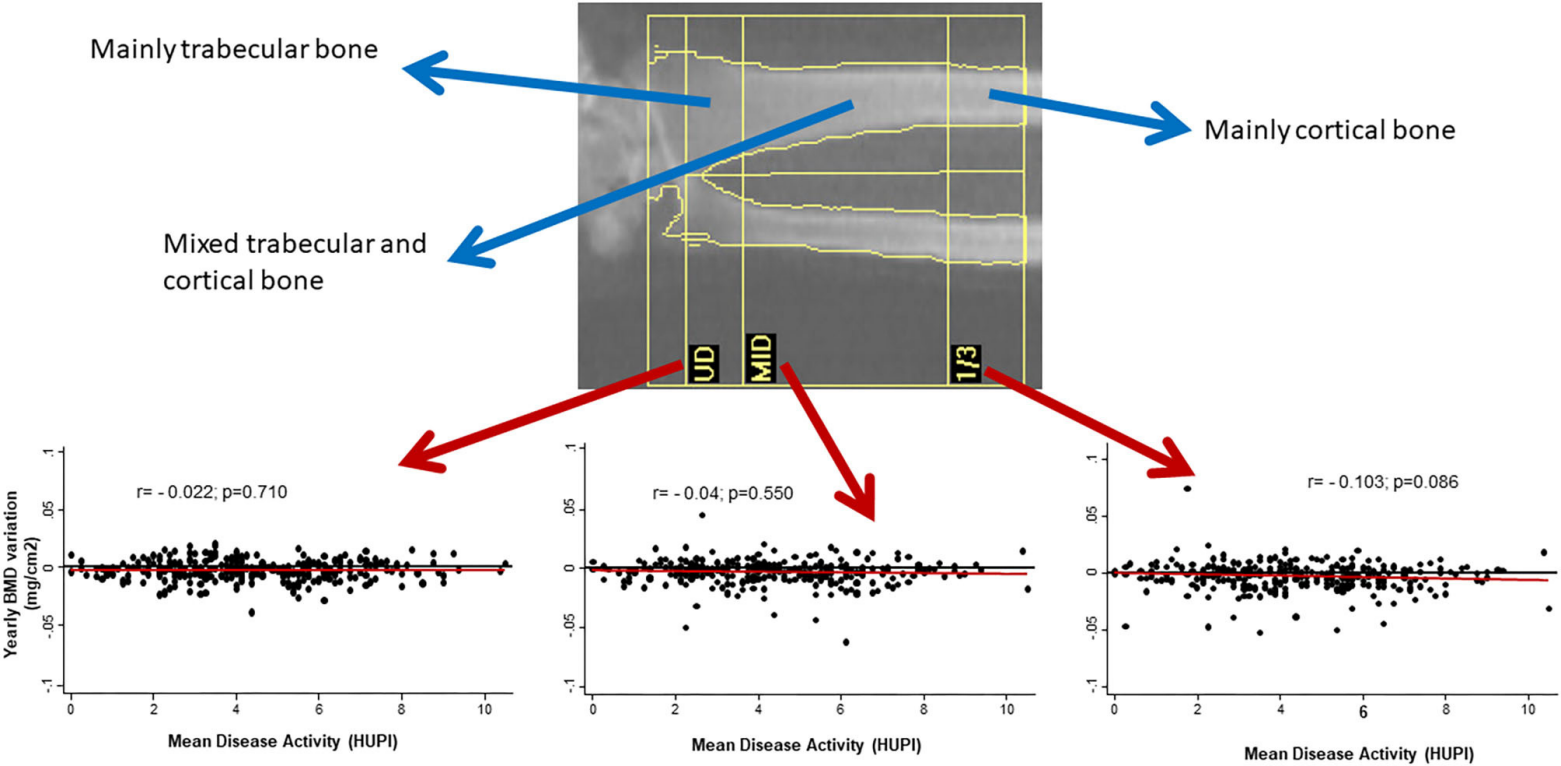
Yearly variation of BMD in mid-radius, but no in other regions of forearm, correlates with cumulative disease activity (assessed by HUPI) after 2 years of follow-up in early arthritis patients. BMD, bone mineral density; UD, ultradistal radius; MID, middle distal radius; 1/3, third distal radius; HUPI, Hospital Universitario de la Princesa index [composite rheumatoid arthritis disease activity index, ref: (12)]; *r*, Pearson's correlation coefficient.

On the other hand, the prevalence of OP in RA is around 30% (up to 50% in post-menopausal women), which might be a two-fold increase over the general population (6, 7). Furthermore, RA patients can experience fractures with higher bone mineral density (BMD) compared to patients without RA (8). In a meta-analysis, the incidence of fragility fractures in RA and general population were 33.00 and 15.31 per 1,000 person-years, respectively (9). The spine is often the most commonly affected site and the incidence of vertebral fractures in RA might be up to 5 times the rate of healthy controls (9, 10). Interestingly, the prevalence of osteopenia and osteoporosis in a large cohort of patients with RA using Vertebral Fracture Assessment (VFA) technique is around 40–60%, while the prevalence of vertebral fractures through VFA images in the same cohort was 13% (11). Paradoxically, in the Princesa Early Arthritis Register Longitudinal (PEARL) study, we observed that cortical bone in mid-forearm seemed to be more susceptible to bone loss than trabecular bone in ultra-distal forearm when disease activity was not adequately controlled (Figure 1).

## Osteoporosis Risk Factors in RA

OP and RA share some common risk factors such as female gender (female: male ratio in RA: 3–4:1) and smoking. Other general OP risk factors such as age, low body mass index (BMI), menopause, diabetes or thyroid disorders (6, 13–15) are equally applicable to patients with RA and to the general population.

Other risk factors that can account for OP in RA include systemic inflammation associated with disease activity, local effect of immune cells leading to bone erosions, glucocorticoid (GC) therapy and impairment of physical activity (14). Therefore, OP and fractures are more frequent in patients with high disease activity (according to DAS28), presence of periarticular bone erosions and cumulative structural damage, RA disease duration ≥ 10 years, high HAQ score or high titers of anti-citrullinated protein antibodies (ACPA) and rheumatoid factor (RF) positivity (6, 9, 10, 14–16). In one study, vertebral fracture risk in RA patients was related to longer disease duration, dose and duration of GC treatment, higher HAQ, Sharp score (cumulative structural damage), ACPA and older age (10). Indeed, ACPA positivity is independently associated with severe trabecular bone loss (13). Furthermore, previous studies have described that the risk of fracture in the next 10 years measured by FRAX is increased in ACPA positive patients (17). By contrast, recent publications show that patients achieving early RA remission have a similar OP risk profile than the general population (15).

Regarding therapeutic agents for RA, GC therapy deserves a special mention. Indeed, GCs suppress osteoblast bone formation, which is associated with a rapid suppression of procollagen type 1 N-terminal pro-peptide (PINP, a biomarker of bone formation), leading to an early reduction in trabecular bone (18). Interestingly, GCs also suppress osteoclast activity, certainly increased in active arthritis patients, which might have a protective effect in some cases (19). In fact, some studies show that GC use in RA could even be beneficial, with a low impact on BMD due to their anti-inflammatory and suppressive effect on arthritis activity (13, 14, 16, 20). Therefore, low doses of GCs could provide protection from inflammatory bone loss during polyarthritis flares and might counteract their unfavorable effects on bone resorption leading to neutral or even positive net skeletal balance (20, 21). The cumulative GC dose (long-term or high dose) as well as the continuous vs. alternative GC dosage strategy are correlated with an increased risk of fracture or a reduced BMD in juxta-articular bone, spine and femoral neck (10, 14, 22, 23). In addition, GCs induce muscle wasting which secondarily increases the risk of falls and fractures (20). However, a daily dose below 5 mg may have a relatively small impact on BMD in RA patients (23).

Besides, the effect of GC therapy is controversial due to limitations in the studies such as concomitant use of anti-resorptive treatment, indication bias of GCs in patients with high disease activity or chronic use of GCs in patients with low disease activity.

Other pharmacological agents involved in fracture risk are opioids, some anti-depressants such as selective serotonin reuptake inhibitors, anti-psychotics, benzodiazepines and proton pump inhibitors (24). The risk related with opioids, higher for vertebral fractures than for non-vertebral fractures, and selective serotonin reuptake inhibitors might be mainly associated with falls (22).

## Bone Homeostasis and Bone Remodeling and the Immune System

**Figure 2:**
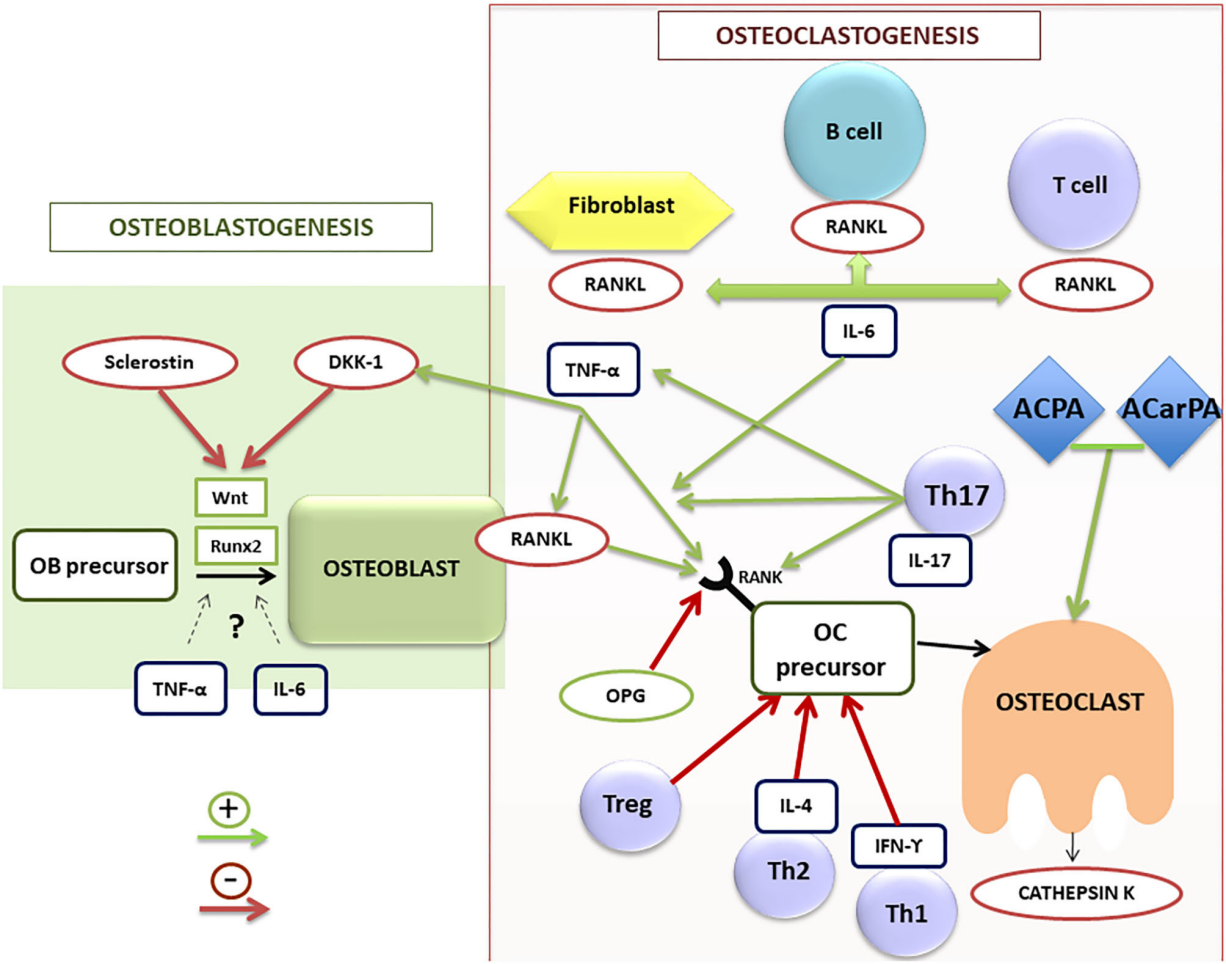
Schematic illustration of osteoblastogenesis and osteoclastogenesis regulation in RA patients. ACPA, anti-citrullinated protein antibodies; ACarPA, anti-carbamylated protein antibodies; DKK-1, dickkopf-1 protein; IL, interleukin family; IFN, interferon; OB, osteoblast; OC, osteoclast; OPG, osteoprotegerin; RA, rheumatoid arthritis; RANK/RANK-L, receptor activator of nuclear factor (NF)-kB (RANK) and its ligand; Runx2, transcription factor involved in the osteoblastogenesis; TNF-α, tumor necrosis factor alpha; Th, T helper lymphocytes; Treg, T regulators lymphocytes.

In the past, the bone seemed to be a static structure, but nowadays is considered a dynamic tissue in constant activity, being the entire skeleton renewed around every 10 years. The dynamic process of bone formation and resorption is known as bone remodeling. This process has a complex regulation, determined by mechanical, molecular and cellular factors. Indeed, osteoclasts, osteoblasts and osteocytes are the main cellular actors involved in bone remodeling. Different signaling pathways of these and other cells of the immune system regulate their function and bone remodeling (25–29) (Figure 2). Indeed, the bone and the immune system maintain a close relationship both anatomical -since the bone houses the bone marrow- and functional through different molecular and cellular signaling pathways and a myriad of cytokines (25, 26, 28, 29). Accordingly, osteoimmunology is a discipline that attempts to address all these interrelations (30) and has undergone a great development in recent years.

RA is the prototype of osteoimmunologic disease where bone loss is one of the most characteristic findings. In RA, there are three kind of bone loss: local, juxta-articular and systemic, causing periarticular osteopenia, bone erosions and generalized osteopenia and/or osteoporosis far from inflamed joints, respectively (25–27). The misbalance in bone remodeling that occurs in RA causes an increase in bone resorption that leads to a reduction of bone mass and a decrease in bone formation that inhibits bone repair. Different cells, cytokines and signaling pathways are involved in both processes and could be potential therapeutic targets to prevent radiographic progression and osteopenia/osteoporosis associated with RA.

### Increased Bone Resorption in RA

Osteoclasts are the main cell population responsible for bone loss in RA patients. These multinucleated large cells are involved in bone resorption through degradation of the bone matrix by acidic and catalytic enzymes, leading to bone erosions in RA (31). They originate from hematopoietic stem cells of the macrophage/monocyte lineage. For their differentiation they require the intervention of the macrophage colony-stimulating factor (M-CSF) (32). In addition, numerous molecules and signaling pathways are involved in the processes of osteoclast differentiation and activation. Among them, the receptor activator of nuclear factor (NF)-kB (RANK) and its ligand (RANKL) are the most important (Figure 2). They are proteins belonging to the TNF superfamily. RANKL is expressed, both soluble and membrane-bound forms, in different bone cells (osteoblasts and osteoclasts) and also in different cell subsets of the immune system (25, 28, 29). By contrast, RANK is mainly expressed in osteoclasts. Osteoprotegerin (OPG) is another protein of the TNF superfamily, which has a regulatory role in bone remodeling (33), working as a RANKL decoy receptor, blocking its effect through its interaction and therefore inhibiting osteoclastogenesis. In fact, RANKL was initially known as the OPG-ligand (34). Therefore, the RANK/RANKL/OPG axis is an essential pathway in the regulation of bone remodeling.

Synovial tissue in RA is the main source of RANKL (35), being synovial fibroblasts and activated T cells the main cells involved in its production (36). Danks et al. found that fibroblasts are the main source of RANKL in synovium, and therefore one of the main responsible for osteoclastogenesis rather than T cells. In fact, in an animal experimental model, the absence of RANKL expression in fibroblasts of mice with collagen-induced arthritis appears to have a protective effect on the appearance of bone erosions. This is not the case for RANKL deficiency in T cells. Therefore, the production of RANKL by fibroblasts could be a possible therapeutic target for the prevention of erosions in RA (37). In addition to RANKL expression, fibroblasts induce osteoclastogenesis through decreasing OPG (38).

T cells also play a crucial role in bone metabolism in RA. As shown in Figure 2, there are different phenotypes of T cells (Th1, Th2, Th17, and Treg), showing patterns of expression of different molecules and of release of distinct cytokines that play different roles in the regulation of bone remodeling. The first two subsets play a negative regulatory role on osteoclastogenesis, secreting inhibitory cytokines such as interferon gamma (IFN-γ) and IL-4 (39). Regulatory T cells (Treg) also work as negative regulators of osteoclastogenesis, being responsible for regulating tolerance and self-reactivity, while Th17 cells are critical stimulators of osteoclastogenesis in RA. Treg cells appear to have an anti-inflammatory action in animal models of TNF-induced arthritis, inhibiting osteoclast differentiation and promoting osteoblast activity (40).

By contrast, Th17 cells have been described as a subtype of Th cells inducing osteoclastogenesis by various mechanisms (41). These cells produce RANKL and IL-17, a cytokine that in turn stimulates RANKL production by fibroblasts and osteoblasts. Furthermore, they stimulate the production of M-CSF and RANKL by osteoblasts and stromal cells, produce TNF-α and increase RANK expression in osteoclast precursors (42). However, although an important role of IL-17, both proinflammatory and osteoclastogenic (43), has been described in RA, treatment with IL-17 inhibitory drugs has not demonstrated clear efficacy in RA patients (44). Therefore, further studies in RA patients are necessary to better define the real role of this cytokine and the profile of patients who could benefit from this targeted therapy. Thus, the balance between Tregs and Th17 cells is crucial for the pathogenesis and the onset of low bone mass and erosions in RA (39, 42).

Finally, B cells, in addition to being antibody-producing cells, also appear to play a role in bone resorption in RA, since they are able to produce RANKL under stimulation. In RA, activated B cells of synovial fluid and peripheral blood have been found to secrete high RANKL levels, thus participating in osteoclastogenesis and bone resorption (45).

In addition to cell subsets, numerous cytokines involved in the pathogenesis of RA have been described, among which TNF-α and IL-6 stand out, because they not only play a role in inflammation, but also seem to have a direct effect on bone remodeling in RA (25–27). In fact, they are two of the main therapeutic targets of the novel RA therapies. Table 1 shows the main cytokines involved in bone destruction and formation in patients with inflammatory diseases.

**Table 1:** Main mediators involved in bone remodeling.

**Pro-bone resorption factors**	**Main effects in bone**
TNF-α	Osteoclast activation. Osteoblast inhibition
IL-6	Osteoclast activation. Osteoblast inhibition
IL-1	Osteoclastogenesis stimulation
IL-8	Osteoclastogenesis stimulation
IL-11	Osteoclastogenesis stimulation
RANKL	Osteoclast activation
IL-17	Osteoclast activation
IL-23	Osteoclast activation
Cathepsin K	Osteoclast activation
M-CSF	Osteoclastogenesis stimulation
**Anti-bone resorption factors**	
IFN-⋎	Osteoclast inhibition
IL-2	Osteoclast inhibition
IL-4	Osteoclast inhibition
OPG	Osteoclast inhibition
**Anti-bone formation factors**	
DKK-1	Osteoblast inhibition
Sclerostin	Osteoblast inhibition
TNF-α	Osteoblast inhibition (dual effect)
IL-6	Osteoblast inhibition (dual effect)

*DKK-1, Dickkopf-1; IFN, interferon; IL, interleukin family; M-CSF, macrophage colony-stimulating factor; OPG, osteoprotegerin; RANKL, receptor activator of nuclear factor (NF)-kB ligand; TNF, tumor necrosis factor*.

TNF-α has a net osteoclastogenic effect. It stimulates bone resorption by promoting osteoclast differentiation, increasing RANKL expression in T and B lymphocytes and osteoclasts, as well as promoting RANK expression in osteoclast precursors (46). TNF-α also contributes to inhibition of bone formation through stimulation of Dickkopf-1 (DDK-1) production (Figure 2). *In vitro* and *in vivo* studies have described a controversial role of TNF-α in osteoblastogenesis, describing both inhibitory (47) and potential promoter effects (48), depending on the stage of the osteoblast differentiation. Accordingly, therapy with TNF-α inhibitors (TNFi) has proven effective not only on inflammation in RA, but also on bone balance, both at the level of systemic bone mass and prevention of radiographic progression (49).

IL-6 is another key cytokine in the pathogenesis of RA (50). In addition to its clear role on inflammation, a direct effect on general and local bone loss has been described in RA (51). IL-6 promotes bone resorption by enhancing the expression of RANKL by osteoblasts, fibroblasts and T cells (52) and is involved in the differentiation of Th17 cells (53). However, IL-6 has a controversial role on bone formation, since both pro-osteoclastogenic (54) and inhibitory (55) roles have been found in *in vitro* studies, depending on the stage of osteoblast precursors. Indeed, therapy with IL-6 inhibitors is effective in controlling inflammation and the radiological progression of RA (56).

Since RANK/RANKL/OPG pathway is crucial in osteoclastogenesis, inhibition of RANKL is a possible therapeutic target to prevent erosions and bone mass loss in RA. In a T cell-dependent animal model of arthritis, blocking RANKL by OPG prevents bone destruction, but not inflammation (57). Furthermore, in a phase II trial to assess the efficacy of denosumab, a RANKL inhibitor human antibody, on several bone parameters in patients with RA, they found that the association of denosumab with methotrexate and other therapies for controlling RA reduces bone erosions, increases BMD and decreases biomarkers of bone resorption, so it could be a potential treatment for erosive RA (58). However, as denosumab has neither an effect on inflammation nor over joint space narrowing, it has not been approved for RA treatment.

Another interesting molecule involved in bone resorption is cathepsin K, a lysosomal cysteine protease expressed predominantly in osteoclasts (59). It is overexpressed in synovial tissue, fibroblasts and serum in RA patients (60). In an animal model of arthritis using human TNF-transgenic mice (hTNF-tg), cathepsin K deficiency inhibits osteoclast activation, preventing joint erosion and presenting a regulatory role on the immune system. Therefore, inhibition of cathepsin K could be a potential adjuvant therapeutic target against bone destruction associated with an inflammatory response (61) if safety issues are finally elucidated.

### Reduced Bone Formation in RA

In the process of bone formation by osteoblasts, cells of mesenchymal origin, different molecules and cell signaling pathways contribute in different ways. One of the most important signaling routes is the Wnt pathway. The name Wnt results from a fusion of the name of the Drosophila segment polarity gene *wingless* and the name of the vertebrate homolog, *integrated* or *int-1*. Wnt signaling pathways are a group of signal transduction pathways which begin with proteins that transduce signals into the cell through cell surface receptors, activating the transcription of genetic factors that regulate osteoblast maturation and therefore bone formation, among other important functions in embryogenesis and organogenesis. There are also different endogenous inhibitors of this pathway, among which DKK-1 and sclerostin are the most important and best known (62). In RA, there is an increase in the expression of these inhibitory factors of the Wnt pathway and therefore a reduction in bone formation. Consequently, this pathway could be involved in the repair of bone erosions in RA (63) (Table 1).

Similarly, an increase in DKK-1 has been reported in patients with RA in inflamed synovium and serum. DKK-1 elevation is associated with an increased risk of erosions in RA patients (64). Its levels seem to depend on the pro-inflammatory state, while inhibiting TNF-α reduces them. Blocking DKK-1 by monoclonal antibodies reduces the occurrence of bone erosions regardless of the inflammatory state in arthritis animal models (65). Therefore, DKK-1 plays an important role in the development of erosions in a pro-inflammatory environment such as RA, and could be a therapeutic target for reducing such erosions.

Sclerostin is another inhibitor of the Wnt pathway, mainly secreted by osteocytes. Blocking sclerostin by a monoclonal antibody in human tumor necrosis factor transgenic (hTNFtg) mice model of arthritis reduces loss of systemic bone mass, periarticular bone destruction and cartilage damage, without any effect on inflammation. The combination of sclerostin and TNF-α inhibition produces normalization of systemic bone mass, inhibits and repairs bone erosions and cartilage damage, thus protecting from structural joint damage (66). However, other studies in animal models of arthritis have not found that sclerostin inhibition decreases or repairs bone erosions or reduces bone loss (67). Therefore, further studies are needed to clarify the role of sclerostin inhibition in RA patients.

## Role of Autoantibodies in Osteoporosis Associated to RA

RA is a systemic inflammatory disease in which the development of different autoantibodies is an early pathogenic event that is associated with structural joint damage, the appearance of erosions and juxta-articular osteopenia (68, 69). The most frequent autoantibodies associated with RA are RF and ACPA. RF is directed against the Fc region of IgG and it mainly appears as an IgM isoform. Although RF is present in a high percentage of RA patients, it is not very specific, since it can appear in other autoimmune diseases and even in healthy population, especially in elderly people. On the contrary, ACPA are more specific of RA and very rare in general population, having demonstrated evidence of their prognostic role on radiological progression and the appearance of erosions. At present, it is well-established that ACPA can be detected in human sera during the pre-RA stage, even 10 years before the onset of symptoms (70).

### RA-Related Autoantibodies as Drivers of Bone Resorption

As already mentioned, the mechanisms by which systemic osteoporosis appears in RA are complex including sustained inflammation, GC use, decrease of physical activity and as a consequence of some disease modifying anti-rheumatic drugs (DMARDs). At present, there is enough evidence to support that autoantibodies play also a role in the pathogenesis of bone loss, either systemic or local, in RA. Different animal models have demonstrated that ACPA can induce osteoclasts differentiation and activation even before arthritis onset (71, 72). During osteoclasts differentiation, the myeloid precursors express citrullinated vimentin in their membrane that can be the target for their specific ACPA. These kind of ACPA attach to Fc-gamma receptor which induces the production of CXCL8 promoting the proliferation and maturation of osteoclasts (71, 72). The presence of immune complexes of ACPA and their targets can also induce this process (73, 74).

Furthermore, Kleyer et al. have demonstrated a decrease in systemic cortical bone mass in a limited population of healthy ACPA-positive subjects without arthritis (75). In this regard, our group has described in patients of an early arthritis cohort with a median symptom duration about 5 months that the ACPA positive subjects showed a significantly lower systemic bone mass at hip and lumbar spine, but not at periarticular level in metacarpophalangeal joints. This effect was independent of the effect of classical risk factors for low bone mass, such as female gender, menopause or BMI (76, 77). Similar data have been described by Bugatti et al. in an untreated early arthritis cohort (78).

In both cohorts, patients had a very short disease duration, with low/no treatment exposure of either GCs or DMARDs, suggesting that ACPA probably have an initial role in the mechanism of BMD loss in these patients. On the other hand, the perpetuation of inflammation and the use of osteoporosis-inducing drugs may collaborate in peripheral BMD loss.

Although the strongest evidence on the role of antibodies as diagnostic and prognostic biomarkers in RA is for ACPA, included as a highly weighted item in the ACR/EULAR criteria for RA classification (79), this role has also been identified for other autoantibodies that recognize post-translational modification of proteins. Among them, anti-carbamylated proteins antibodies (anti-CarPA) have the strongest evidence regarding their role in the pathogenesis of RA compared to anti-acetylated proteins or other modifications. Interestingly, many of these modifications (citrullination, acetylation or carbamylation) include vimentin, pointing to this protein as an important target in RA pathogenesis. Anti-CarPA have shown a clear overlap with ACPA, but some studies have identified them as an independent prognostic biomarker, especially regarding the appearance of erosions (80).

The role of anti-CarPA in BMD has also been studied in early arthritis cohorts. Regueiro et al. described that high titers of anti-CarPA were associated with lower systemic BMD, either at lumbar spine or hip, in these patients, but not at local level in metacarpophalageal joints, and this association was independent of ACPA titers (81).

As mentioned above, the sustained presence of inflammatory cytokines in RA plays an important role in regulating osteoclast activation. But there is also evidence of the importance of autoantibodies and immune complexes for the production of these inflammatory cytokines by macrophages, showing in an indirect way how these immune complexes contribute to bone mass loss through regulation of osteoclasts.

All together, these data suggest that ACPA probably have an initial role in systemic osteoporosis in the earliest moments of the disease, and lead to local BMD loss in later stages through the perpetuation of inflammation and the progressive increase in their titers.

## Bone Mineral Density as Possible Severity Marker in RA

Currently, the diagnostic and therapeutic strategies aim at the early detection and treatment of the disease (82). Indeed, in the PEARL study the implementation of early DMARD treatment in tight control and treat to target strategies have led to prevention of erosive disease and arrest of radiological progression (83), both due to a better control of the disease and a reduced use of long-term osteopenizing drugs. Furthermore, the decrease of autoantibody titers has probably also contributed to a lower loss of both systemic and local BMD. Therefore, it is important to have prognostic biomarkers that help us better understand disease evolution and detect and treat these patients early and correctly to avoid long-term comorbidities.

The association of RA-related autoantibodies with worse BMD suggests that measurement of bone mass could help to predict prognosis of patients with early arthritis. Regarding this topic, there is evidence that measurement of BMD by dual X-ray radiogrammetry (DXR) at metacarpal diaphysis in the non-dominant hand of RA patients is associated with disease progression, appearance of bone erosions and even, in some studies, with increased mortality (84, 85). In addition, DXR is a very sensitive procedure to detect loss of BMD in the hand, which in long-standing RA has been associated with high titers of autoantibodies, mainly ACPA, radiographic progression and the appearance of erosions (86).

However, DXR is a poorly accessible and expensive technique. On the contrary, conventional dual X-ray densitometry (DXA) is the gold standard for assessing BMD in OP, and is also a simple and more accessible technique all over the world. Of note, our group has recently proved that measurement of BMD at metacarpal diaphysis with conventional DXA is reproducible and closely correlates with DXR measurements (87, 88).

## Something Is Changing in Osteoporosis Associated With RA

Interestingly, a decrease in the prevalence of OP and fractures has been described in the last 10 years, likely due to improved therapeutic options that have allowed rheumatologists to lead more RA patients to remission (15).

Most of the information comes from TNFi, which have been associated with a reduced number of fractures and improvement of BMD in both vertebral and non-vertebral anatomical locations (22, 89, 90). Regarding bone turnover markers, the results of the studies were quite consistent, often showing an increase in bone formation markers along with a decrease in bone resorption ones (89–92). Less often, they showed either a decrease in bone resorption with stabilization of bone formation (93, 94), or stabilization of bone resorption and increased formation (95, 96). A recent paper showed, in patients with early RA treated with TNFi (certolizumab pegol), that bone turnover markers and Wnt/B-catenin pathway inhibitors may change quickly after starting therapy, with a decrease in carboxy -terminal telopeptide of type 1 collagen (CTX-I), an increase in PINP and the decrease in DKK-1 and sclerostin, already evident from the first week of therapy (92).

Other biological agents such as tocilizumab, rituximab, and abatacept have shown a significant decrease in bone resorption markers and RANKL expression, which provides evidence of a beneficial effect on bone remodeling process slowing down bone loss (15). In a study, a 2-year treatment with tocilizumab showed improvement in BMD and significantly decreased levels of β-CTX in ACPA positive patients (97). Another study disclosed the efficacy of abatacept for increasing BMD at the femoral neck without differences in urinary levels of cross-linked N-telopeptide of type I collagen (uNTx) and bone-specific alkaline phosphatase (98).

Finally, most information about DMARDs reflects a non-deleterious effect on BMD, especially with methotrexate (99), although in another study, leflunomide was the only DMARD associated with significant increase in lumbar spine BMD without differences in femoral neck (100).

All together, this evidence suggests that the relationship between RA disease activity, systemic inflammation and OP is mediated by pro-inflammatory cytokines (mainly M-CSF, IL-17, TNF-α, IL-1, and IL-6) that regulate osteoclastogenesis and are important stimulators of RANKL synthesis. During the inflammatory process, their production exceeds the synthesis of their physiologic inhibitors and decoy receptor OPG. Therefore, the imbalance between osteoblast and osteoclast activity is directly responsible for bone loss and local erosions in RA (15, 16). Nevertheless, this imbalance can also occur at systemic level. This fact posits that BMD loss in RA may be an early predictor of erosive disease (16), as suggested by a study in which BMD loss at the metacarpal site was the main independent predictor of subsequent articular radiographic progression (14).

## Conclusions

OP is a common complication of RA patients, mainly due to shared demographic characteristics as well as common pathogenic pathways to both entities. The mechanisms involved in the pathogenesis of RA-associated OP are complex. It is evident that the RANK/RANKL/OPG and the Wnt/DKK-1/sclerostin pathways play a crucial role in the development of systemic and local OP as well as bone erosions. Furthermore, different pro-inflammatory cytokines involved in RA pathogenesis such as TNF-α, IL-6 and IL-17, among others, have a relevant role in the regulation of bone homeostasis. Despite these mechanisms are complex and controversial, targeting these molecules clearly provides a drastic arrest of radiographic progression as well as an improvement of disability and quality of life of RA patients. Recently, some studies suggest that this strategy also reduces OP and fractures, further improving the clinical outcome of these patients, especially in long-term disease.

Regarding the role of autoantibodies, both ACPA and anti-CarPA have shown a pathogenic role affecting bone homeostasis through their involvement in the development of systemic OP and bone erosions. These autoantibodies also allow classifying different phenotypes of RA patients regarding evolution and prognosis. Some doubts that need to be clarified still remain, such as the duality of some inflammatory molecules and their involvement in bone homeostasis in RA. There are also unmet needs like clinical studies that correlate these findings and identify prognostic factors capable of helping in decision-making and in the monitoring and treatment of these patients.

Finally, we must be optimistic as biological therapies developed in recent years make it possible to reverse part of the negative effects of RA on bone and even seem to reduce the risk of osteoporotic fractures.

## Author Contributions

All the authors have contributed to the design, interpretation of data, writing, and supervision of the final version of this manuscript.

## Conflict of Interest

The authors declare that the research was conducted in the absence of any commercial or financial relationships that could be construed as a potential conflict of interest.

## Abbreviations

ACPA: anti-citrullinated protein antibodies
ACR: American College of Rheumatology
Anti-CarPA: anti-carbamylated protein antibodies
BMD: bone mineral density
BMI: body mass index
CI: confidence interval
CTX-I: carboxy-terminal telopeptide of type 1 collagen
CXCL8: C-X-C motif chemokine ligand 8
DAS28: disease activity score counted over 28 joints
DDK-1: Dickkopf-1 protein
DMARD(s): disease modifying anti-rheumatic drug(s)
DXA: dual X-ray densitometry
DXR: dual X-ray radiogrammetry
EULAR: European league against Rheumatism
FRAX: fracture risk assessment tool
GC(s): glucocorticoid(s)
HAQ: health assessment questionnaire
hTNF-tg: human tumor necrosis factor transgenic
Ig: immunoglobulins
IL: interleukin
IFN-⋎: interferon gamma
M-CSF: macrophage colony-stimulating factor
OP: osteoporosis
OPG: osteoprotegerin
PEARL: Princesa early arthritis register longitudinal
PINP: procollagen type 1 N-terminal propeptide
RA: rheumatoid arthritis
RANK/RANK-L: receptor activator of nuclear factor (NF)-kB (RANK) and its ligand
RF: rheumatoid factor
TNF-α: tumor necrosis factor
TNFi: TNF-α inhibitors
Th: T helper lymphocytes
Treg: regulatory T cells
uNTx: cross-linked N-telopeptide of type I collagen
Wnt: drosophila segment polarity gene *wingless* and *integrated* or *int-1* of the vertebrate homolog.
